# A Silica Symptom Verified

**Published:** 1892-12

**Authors:** E. Rushmore

**Affiliations:** Plainfield, N. J.


E. Rushmore, M. D., Plainfield, N. J.

### A SILICA SYMPTOM VERIFIED.

E. Rushmore, M. D., Plainfield, N. J.

A lady with a scirrhous tumor of the left mamma being referred to me for treatment, I found among other symptoms, this peculiar one: dryness felt in the tips of the fingers as if made of paper, at night. She was also subject to sharp stinging pains in the tumor. The symptom of the finger tips was found only under Ant-t. and Silica; occurring in the afternoon under Silica only. It proved a decisive and trustworthy indication, as the first dose of Silica removed the dryness and she spoke of the Silica as also acting like a charm on the pain in the breast.

